# Computational Methods for Estimation of Cell Cycle Phase Distributions of Yeast Cells

**DOI:** 10.1155/2007/46150

**Published:** 2007-08-19

**Authors:** Antti Niemistö, Matti Nykter, Tommi Aho, Henna Jalovaara, Kalle Marjanen, Miika Ahdesmäki, Pekka Ruusuvuori, Mikko Tiainen, Marja-Leena Linne, Olli Yli-Harja

**Affiliations:** 1 Institute of Signal Processing, Tampere University of Technology, P.O. Box 553, Tampere 33101, Finland; 2 MediCel Ltd., Haartmaninkatu 8, Helsinki 00290, Finland

## Abstract

Two computational methods for estimating the cell cycle phase distribution of a budding yeast *(Saccharomyces cerevisiae)* cell population are presented. The first one is a nonparametric method that is based on the analysis of DNA content in the individual cells of the population. The DNA content is measured with a fluorescence-activated cell sorter (FACS). The second method is based on budding index analysis. An automated image analysis method is presented for the task of detecting the cells and buds. The proposed methods can be used to obtain quantitative information on the cell cycle phase distribution of a budding yeast *S. cerevisiae* population. They therefore provide a solid basis for obtaining the complementary information needed in deconvolution of gene expression data. As a case study, both methods are tested with data that were obtained in a time series experiment with *S. cerevisiae*. The details of the time series experiment as well as the image and FACS data obtained in the experiment can be found in the online additional material at http://www.cs.tut.fi/sgn/csb/yeastdistrib/.

## 

[1,2,3,4,5,6,7,8,9,10,11,12,13,14,15,16,17]
